# Clinical Safety Profile of Bruton’s Tyrosine Kinase (BTK) Inhibitors in Chronic Lymphocytic Leukaemia: A Real-World Ambispective Study From India

**DOI:** 10.7759/cureus.109238

**Published:** 2026-05-19

**Authors:** Arya Pradhan, Harsha P Panchal

**Affiliations:** 1 Department of Medical Oncology, The Gujarat Cancer and Research Institute, Ahmedabad, IND

**Keywords:** acalabrutinib, bruton’s tyrosine kinase inhibitors, chronic lymphocytic leukaemia, ibrutinib, lymphocytes

## Abstract

Background: Chronic lymphocytic leukaemia (CLL) is the most common leukaemia among adults in Western regions, while India reports comparatively lower incidence rates. Bruton’s tyrosine kinase (BTK) inhibitors, particularly ibrutinib and acalabrutinib, have reshaped CLL therapy by blocking B-cell receptor signalling pathways essential for malignant B-cell survival.

Objective: Given the increasing use of BTK inhibitors in routine practice and the limited real-world Indian data, this study evaluates the clinico-safety profile of BTK inhibitors and identifies predictors of response and progression in a pragmatic clinical setting.

Materials and methods: This was a single-centre ambispective observational study conducted from June 2021 to June 2024 in patients diagnosed with CLL aged 18-70 years. The patients received ibrutinib (420 mg once daily) or acalabrutinib as per availability and physician decision.

Results: Among the 93 patients included, most were male and younger than typical Western CLL cohorts. Hematologic recovery rates were high, with normalisation of haemoglobin, platelet count, and leukocyte count seen in the majority within four to seven months of therapy. The overall median progression-free survival (PFS) was 14 months.

Conclusion: BTK inhibitors have significantly improved therapeutic outcomes in CLL. Both ibrutinib and acalabrutinib demonstrated meaningful hematologic responses and prolonged disease control. Acalabrutinib showed superior tolerability and better treatment adherence. Expanding access to genomic testing and improving drug availability will be essential to optimise CLL care in India.

## Introduction

Chronic lymphocytic leukaemia (CLL) is a clinically heterogeneous haematological malignancy characterised by the progressive clonal expansion and accumulation of functionally incompetent mature B lymphocytes in the peripheral blood, bone marrow, and lymphoid tissues. It is the most common adult leukaemia in Western populations and demonstrates marked variability in disease course and treatment outcomes [1].

Despite a lower reported incidence of CLL in India and other Asian countries, Indian patients often present at a younger age with more advanced disease and adverse biological features, suggesting possible regional and ethnic heterogeneity in disease biology and clinical outcomes. Indian cohorts have reported a median age at presentation of approximately 55-60 years, nearly a decade younger than that observed in Western populations. Differences in baseline disease characteristics, comorbidity burden, treatment accessibility, and affordability may further influence therapeutic outcomes in routine clinical practice.

The clinical course of CLL is strongly influenced by cytogenetic and molecular abnormalities, such as 17p deletion and TP53 mutations, which are associated with resistance to conventional chemoimmunotherapy and poorer survival [2]. The advent of targeted therapies, particularly Bruton’s tyrosine kinase (BTK) inhibitors, has significantly transformed CLL management by disrupting B-cell receptor signalling pathways essential for malignant B-cell survival and proliferation [3].

Ibrutinib, the first-in-class irreversible BTK inhibitor, demonstrated substantial improvements in progression-free and overall survival (OS) across treatment-naïve and relapsed/refractory CLL populations, including patients with high-risk genetic features [4]. However, off-target kinase inhibition with ibrutinib has been associated with adverse events such as atrial fibrillation, bleeding, and hypertension, which may affect long-term tolerability and treatment adherence. These limitations led to the development of second-generation BTK inhibitors such as acalabrutinib, which offer greater kinase selectivity, comparable efficacy, and an improved safety profile [5,6].

With the increasing incorporation of BTK inhibitors into routine clinical practice, real-world evidence is essential to complement clinical trial data, particularly in underrepresented populations such as Indian patients. However, Indian real-world data evaluating the clinical safety profile, effectiveness, and predictors of response and progression with BTK inhibitors remains limited [7]. Therefore, this study aims to assess the effectiveness and safety of BTK inhibitors in a pragmatic clinical setting and to identify factors associated with treatment response and disease progression.

Real-world evidence is important because outcomes observed in controlled clinical trials may not fully reflect routine clinical practice. Indian patients often differ in demographic characteristics, comorbidity profiles, treatment accessibility, and affordability, making real-world evaluation of BTK inhibitors essential for understanding their effectiveness and safety in pragmatic settings.

## Materials and methods

Study design

**Figure 1:**
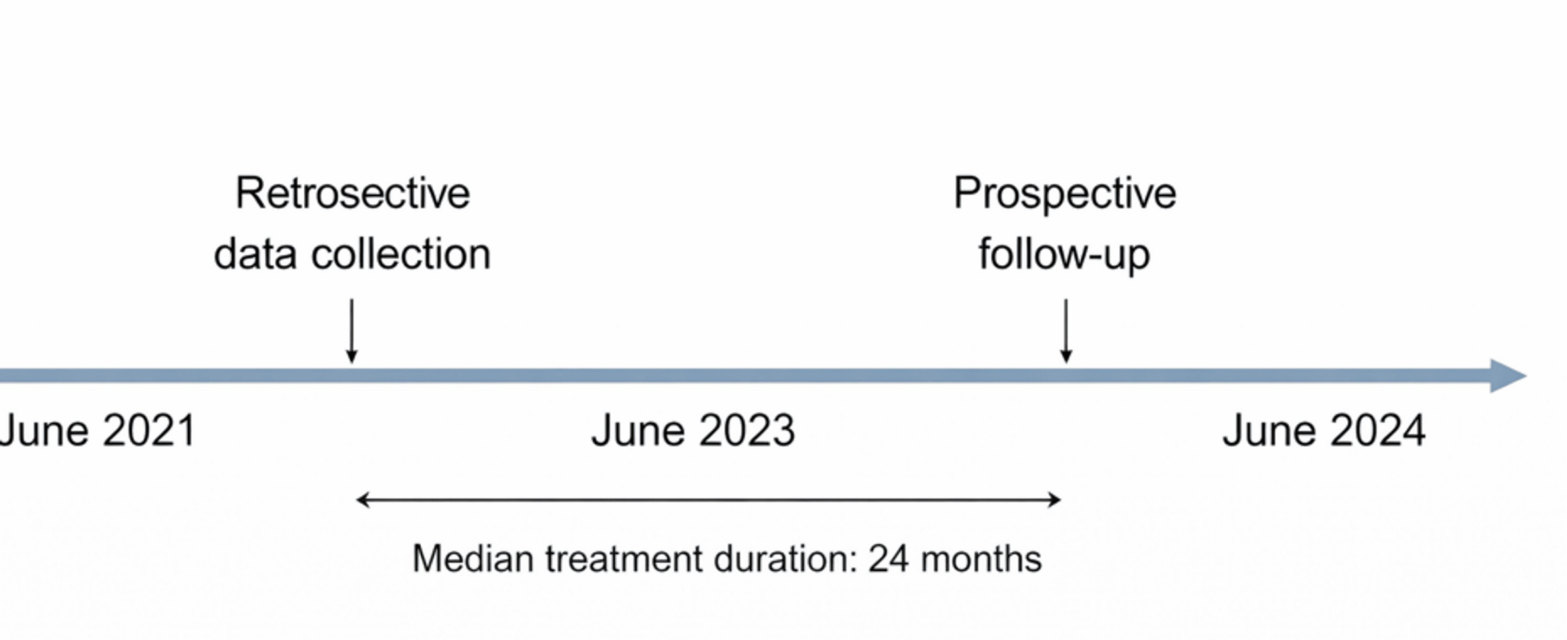
Study timeline of the ambispective cohort. The study spanned from June 2021 to June 2024, comprising a retrospective data collection phase from June 2021 to June 2023, followed by a prospective follow-up phase until June 2024. The median treatment duration was 24 months. Image Credit: The image was created by the authors using Microsoft PowerPoint (Microsoft Corp., Redmond, WA, USA).

**Figure 2:**
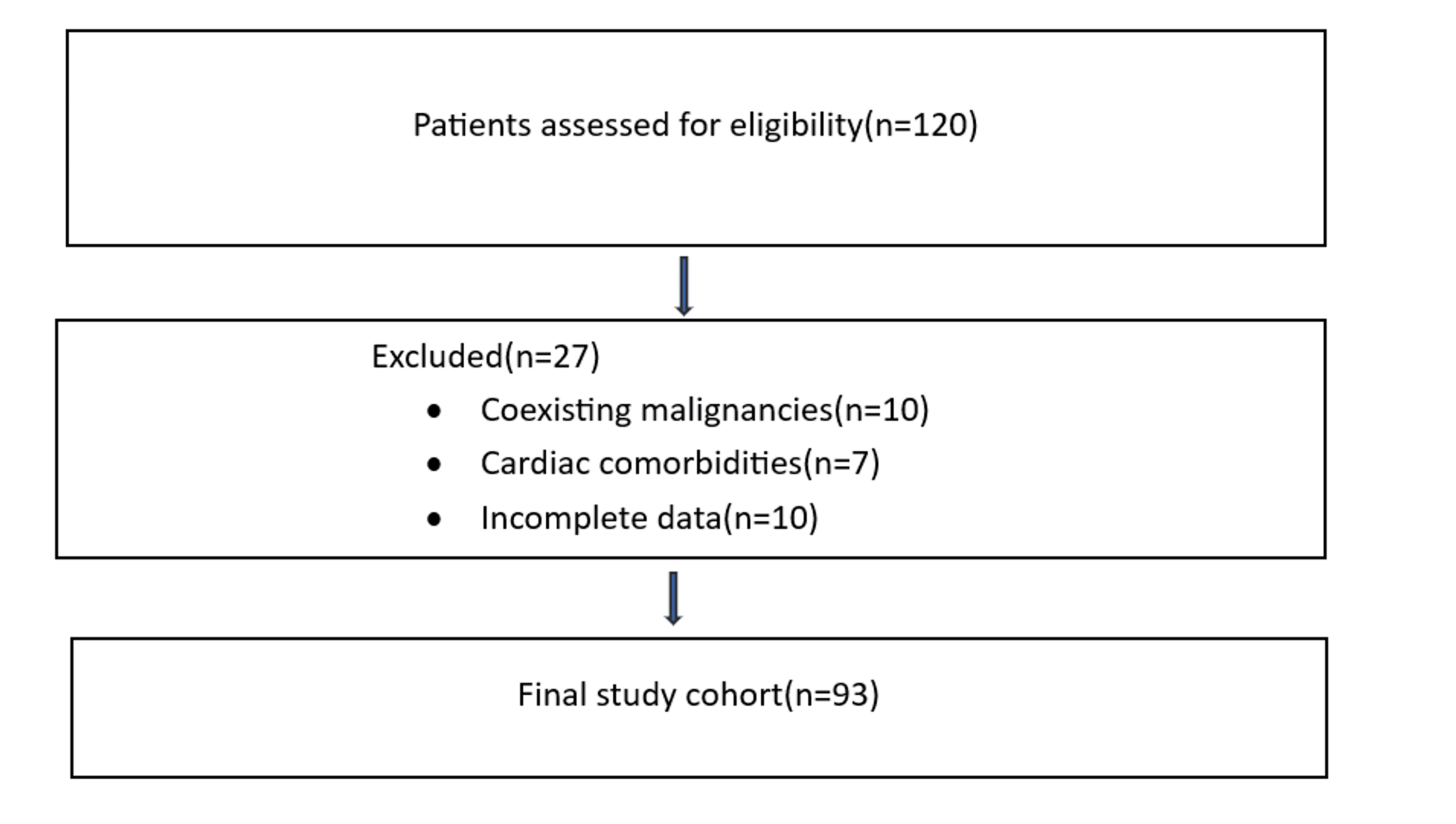
Flowchart of patient selection. A total of 120 patients with chronic lymphocytic leukaemia (CLL) were assessed for eligibility. Twenty-seven patients were excluded due to coexisting malignancies (n = 10), cardiac comorbidities (n = 7), and incomplete data (n = 10). The final study cohort consisted of 93 patients. Image Credit: The flowchart was created by the authors using Microsoft Word (Microsoft Corp., Redmond, WA, USA).

This was a single-centre, ambispective, observational study conducted between June 2021 and June 2024. Adult patients aged ≥18 years with a confirmed diagnosis of CLL were eligible for inclusion. All enrolled patients had an Eastern Cooperative Oncology Group (ECOG) performance status of 0-2 and adequate baseline renal, hepatic, and hematologic parameters. Patients with coexisting malignancies, documented bleeding disorders, or significant cardiac or hepatic comorbidities that could interfere with treatment safety assessment or study outcomes were excluded. This study included all consecutive eligible patients with CLL who received BTK inhibitor therapy during the study period at our institution. As this was a retrospective ambispective observational cohort study, a formal sample size calculation was not performed. The ambispective study design and follow-up timeline are illustrated in Figure 1. The patient screening and cohort selection process is summarised in Figure 2.

Study population

This ambispective observational cohort study included patients diagnosed with CLL who received BTK inhibitor therapy at our centre during the study period. Both treatment-naïve and relapsed/refractory patients were included.

Patients who had already initiated BTK inhibitor therapy prior to enrollment, as well as patients newly started on treatment during the study period, were eligible for inclusion. Treatment selection between ibrutinib and acalabrutinib was based on physician discretion, patient affordability, drug availability, and clinical considerations. Patients with incomplete treatment records or inadequate follow-up data were excluded from the analysis. Clinical characteristics, treatment response, adverse events, treatment interruptions, dose modifications, and progression-free survival (PFS) were evaluated using available medical records and follow-up data.

Patients diagnosed with CLL were identified from institutional records. A consecutive sampling technique was employed, wherein all patients fulfilling the inclusion criteria during the study period were included.

The primary objective of the study was to evaluate the real-world effectiveness and safety profile of BTK inhibitors in patients with CLL, including assessment of hematologic response, adverse events, treatment adherence, and PFS. The secondary objective was to descriptively compare the clinical outcomes and tolerability profiles of ibrutinib and acalabrutinib in routine clinical practice.

Inclusion criteria

Patients diagnosed with CLL who received treatment with BTK inhibitors during the study period and had adequate clinical records available for analysis.

Exclusion criteria

Patients with coexisting malignancies, significant cardiac comorbidities, incomplete clinical data, or those lost to follow-up were excluded from the study.

Data selection process

Patient data were retrieved from institutional records. Records were screened based on diagnosis, treatment details, and data completeness. Patients meeting the inclusion criteria were selected, while those with missing or inadequate data and predefined exclusion conditions were systematically excluded to ensure data reliability and consistency. This ambispective observational cohort study was reported in accordance with the Strengthening the Reporting of Observational Studies in Epidemiology (STROBE) guidelines.

Scores and scales

Clinical status was assessed using the ECOG Performance Status [1]. Disease staging was determined according to the Rai staging system [2]. Adverse events were graded according to the National Cancer Institute Common Terminology Criteria for Adverse Events (CTCAE) version 4.03, as per the institutional protocol followed during the study period [3]. All scoring systems were applied as per standard definitions, and appropriate references were cited. No additional permissions were required for the use of these standardised clinical scales.

Patients and treatment

Patients received either ibrutinib or acalabrutinib based on physician discretion, drug availability, clinical profile, and affordability. Ibrutinib was administered at a dose of 420 mg once daily, while acalabrutinib was administered at a dose of 100 mg twice daily. The average duration of treatment was 24 months (range: 2 to 36 months). Dose modifications due to toxicities were necessary in 12% of patients.

Study assessments

Data collection included demographic characteristics, clinical course of the disease, baseline and post-treatment hematologic parameters, and documented adverse events. Treatment-related toxicities were graded according to the CTCAE version 4.03. Treatment response was assessed according to the International Workshop on Chronic Lymphocytic Leukaemia (iwCLL) criteria. Complete response (CR), partial response (PR), stable disease (SD), and progressive disease (PD) were defined as per standard iwCLL recommendations. Transient treatment-related lymphocytosis occurring during early BTK inhibitor therapy was not considered disease progression unless accompanied by other clinical or radiological evidence of progression.

Statistical analysis

The sample size was estimated using the formula: $n = \frac{Z^{2} \times p \times (1 - p)}{d^{2}}$, where n is the sample size, Z is the standard normal deviate (1.96 for 95% confidence level), p is the expected proportion, and d is the margin of error. Due to the retrospective nature of the study, all eligible patients meeting the inclusion criteria during the study period were included.

Statistical analysis was performed using IBM SPSS Statistics for Windows, Version 29 (Released 2022; IBM Corp., Armonk, New York, United States). Continuous variables were summarised using mean ± standard deviation or median (interquartile range), while categorical variables were expressed as frequencies and percentages. Appropriate statistical tests (e.g., chi-square test, t-test) were applied, with a p-value <0.05 considered statistically significant. PFS was estimated using the Kaplan-Meier method and compared using the log-rank test where applicable. Patients without progression or death at the last follow-up were censored on the date of the last clinical evaluation. Cox proportional hazards regression analysis was performed for exploratory assessment of predictors of progression where relevant.

## Results

Patient demographics and baseline characteristics

A total of 93 patients with CLL were included in the study. The median age of the patients was 57 years (range: 46-72 years). The majority of the patients were male (78%, n = 73), while females comprised 21.5% (n = 20). Most patients had a baseline ECOG performance status of 0-1 (89.2%, n = 83), while the remaining patients had a performance status greater than 1. Additional demographic and clinical characteristics are summarised in Table 1.

**Table 1:** Baseline characteristics of the study population. Data are presented as numbers (percentages) for categorical variables and median (interquartile range) for continuous variables. No comparative statistical tests were applied, as this table describes baseline characteristics. ECOG PS: Eastern Cooperative Oncology Group performance status; IQR: interquartile range; HBV: hepatitis B virus

Parameter	Value (N = 93)
Median age (IQR)	57 years (46-72)
Gender (male/female)	73 (78.5%)/20 (21.5%)
ECOG PS 0-1	83 (89.2%)
Hypertension	18 (19.4%)
Type 2 diabetes mellitus	10 (10.8%)
HBV titres seropositivity (latent)	4 (4.3%)
Median disease duration	24 months

Subgroup analysis

Subgroup analyses were performed to explore associations between selected clinical variables and PFS. Patients who achieved early hematologic normalisation within four months demonstrated a numerically longer median PFS (18 months) compared with those with delayed normalisation (12 months); however, this difference did not reach statistical significance (p = 0.07).

Patients aged ≤60 years had a median PFS of 16 months compared with 13 months among patients aged >60 years. This difference was not statistically significant (p=0.12). No significant difference in PFS was observed between patients with total leukocyte count (TLC) ≤100,000 and >100,000 (p = 0.96). Patients with ECOG performance status 0-1 had a median PFS of 15 months compared with 12 months among patients with ECOG performance status >1; however, this finding was also not statistically significant (p = 0.08). Given the limited sample size and relatively short follow-up duration, these subgroup findings should be interpreted cautiously.

**Figure 3:**
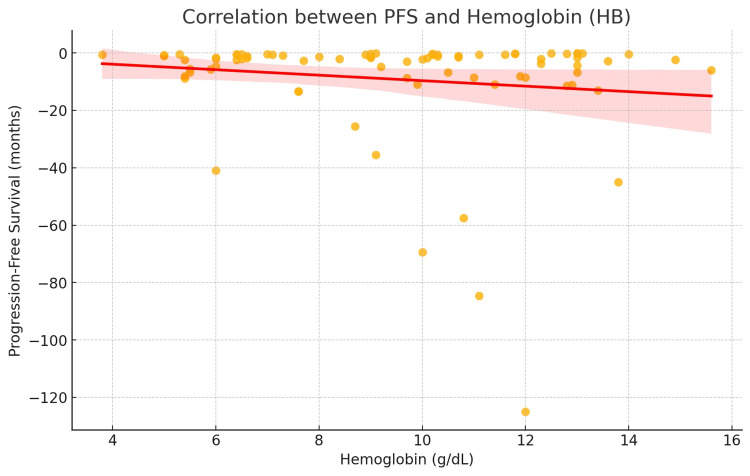
Correlation between progression-free survival (PFS) and haemoglobin (HB) levels, along with a regression line.

**Figure 4:**
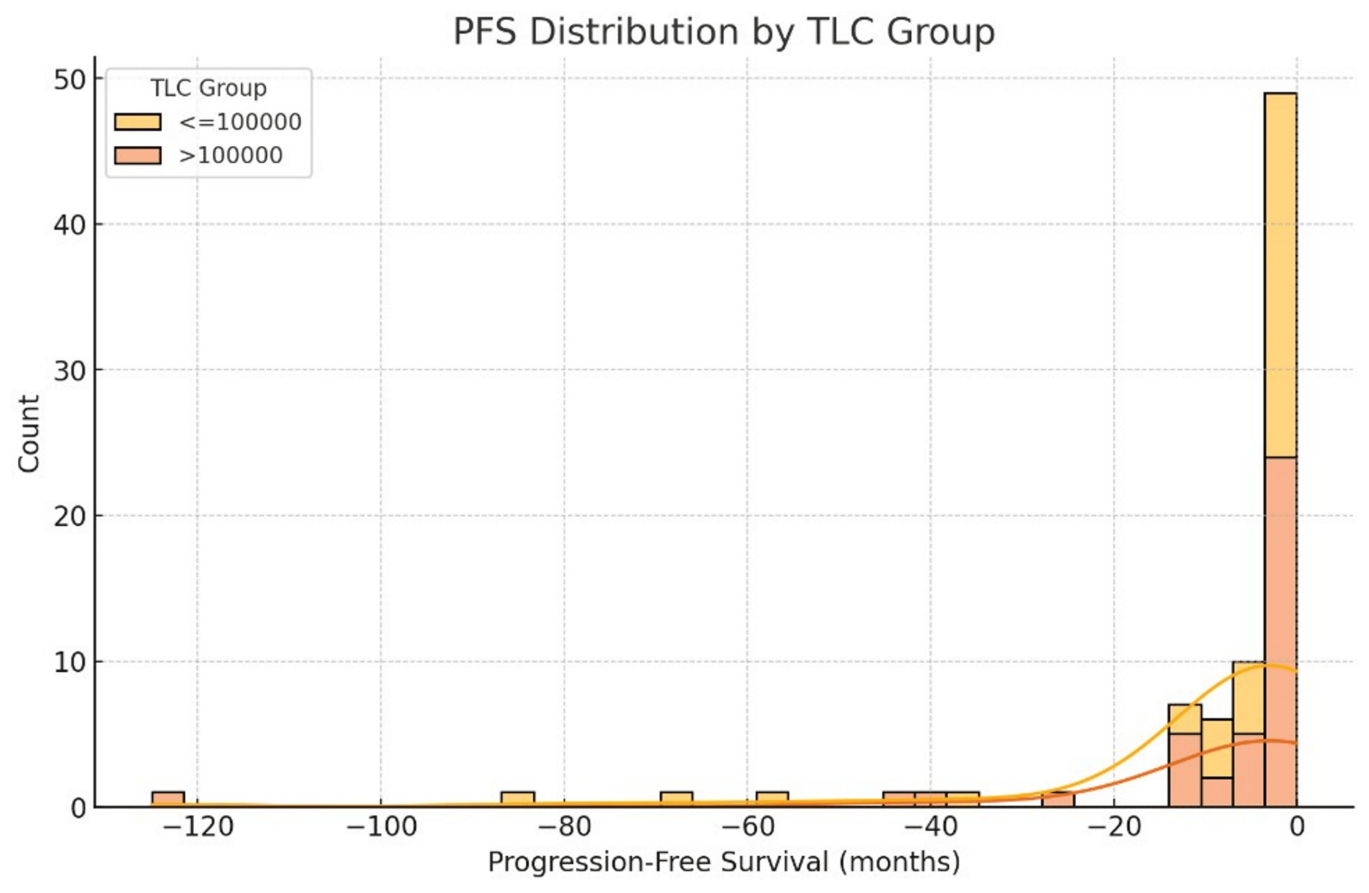
Progression-free survival (PFS) distribution among patients with total leukocyte count (TLC) ≤100,000 and >100,000.

**Figure 5:**
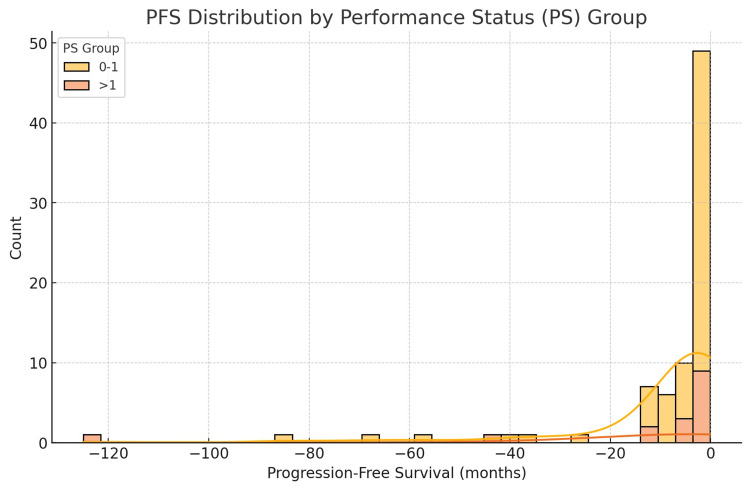
Progression-free survival (PFS) distribution among patients aged ≤60 years and >60 years.

A scatter plot shows a weak negative correlation between HB and PFS, with a correlation coefficient of -0.14, as shown in Figure 3. This suggests that higher HB levels do not correlate strongly with prolonged PFS in this cohort. OS data and time to next treatment (TTNT) were not part of this analysis but should be explored in future follow-ups to better understand long-term efficacy. The subgroup analysis of PFS in association with TLC and age group is shown in Figures 4-5, respectively.

**Figure 6:**
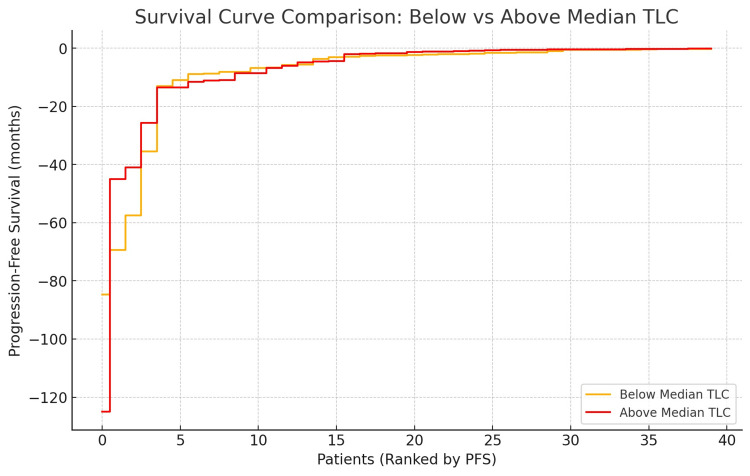
Progression-free survival (PFS) distribution between two patient groups based on their total leukocyte count (TLC).

PFS between two patient groups based on their TLC at presentation, as shown in Figure 6. Patients were stratified into two groups based on the median TLC value: the below-median TLC group (yellow line), comprising patients with TLC values below the calculated median, and the above-median TLC group (red line), comprising patients with TLC values above the median.

**Figure 7:**
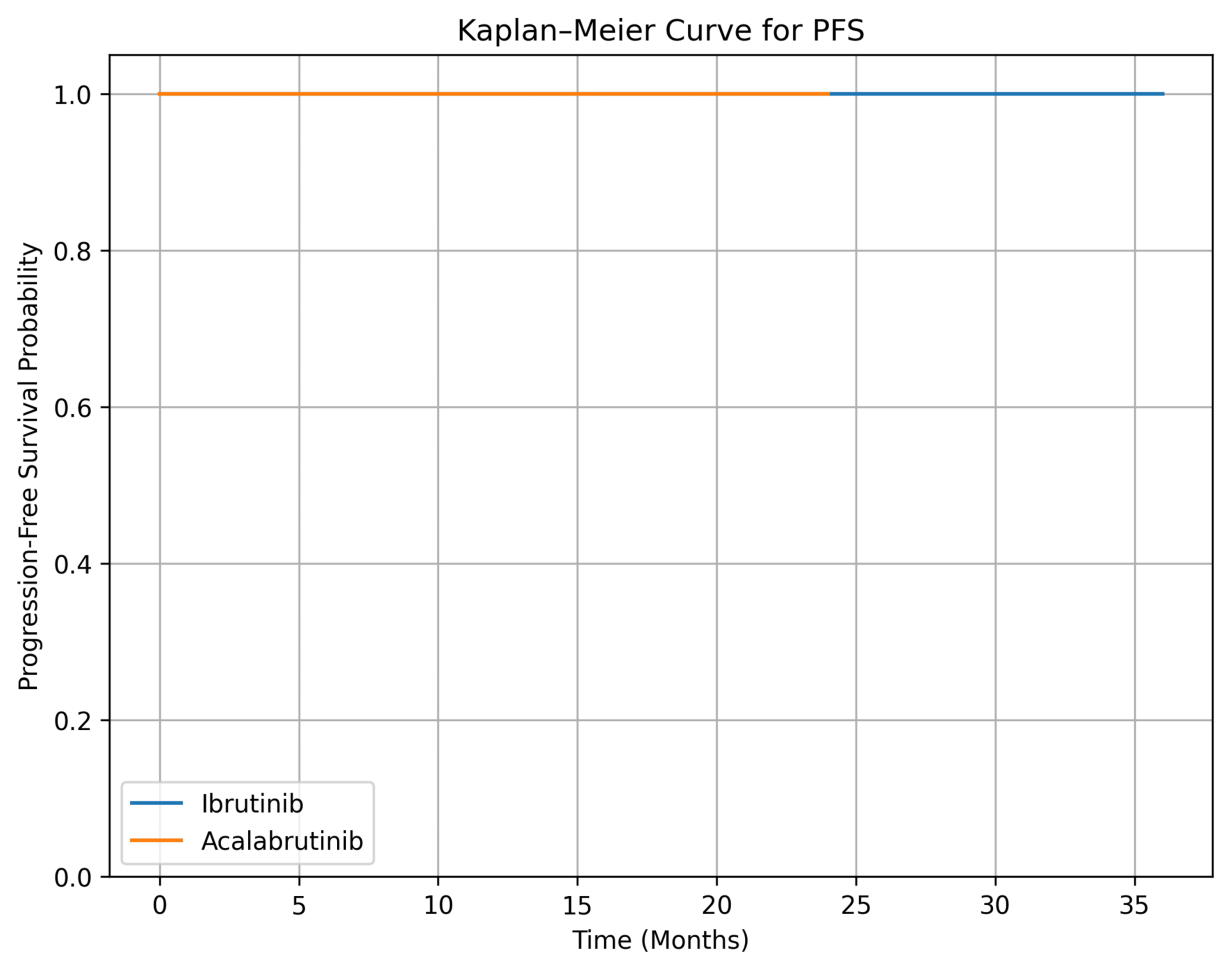
Kaplan-Meier curve comparing progression-free survival (PFS) between ibrutinib and acalabrutinib.

Kaplan-Meier analysis demonstrated largely overlapping survival curves between treatment groups, suggesting no significant difference in PFS during the available follow-up period. The limited number of progression events and relatively short follow-up duration may have reduced the ability to detect meaningful differences between subgroups (Figure 7).

**Figure 8:**
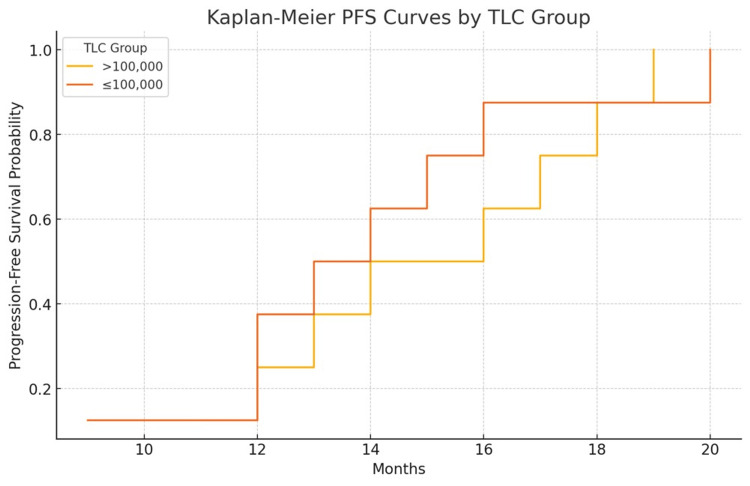
Progression-free survival (PFS) among patients with total leukocyte count (TLC) ≤100,000 and those with TLC >100,000.

The plot in Figure 8 compares PFS based on TLC, divided into two groups: patients with TLC ≤100,000 and those with TLC >100,000. The curve demonstrates how leukocyte count at presentation may impact the progression and treatment outcomes in patients.

**Figure 9:**
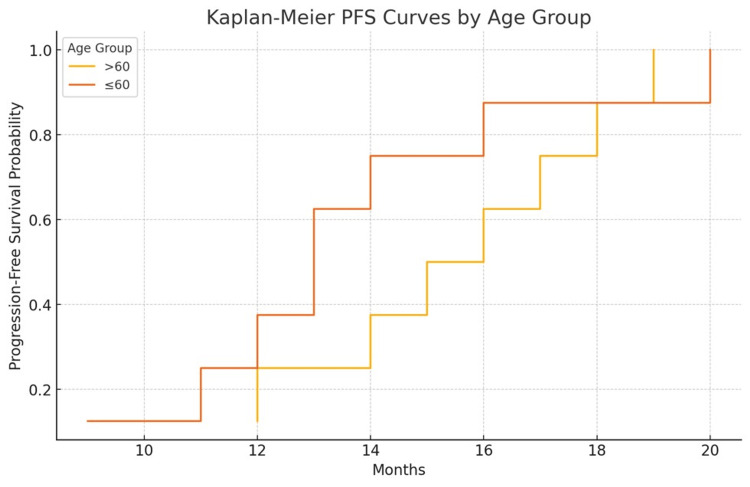
Progression-free survival (PFS) among patients aged ≤60 years versus those older than 60 years.

The stepwise progression of the curve, as shown in Figure 9, highlights how each group progresses over time, with potential differences in survival probabilities depending on age.

The most commonly observed adverse events included anaemia (6.5%), neutropenia (6.5%), arthralgia (6.5%), thrombocytopenia (5.4%), hypertension (4.3%), fatigue (4.3%), and infections (4.3%). Atrial fibrillation/flutter was observed in two patients (2.2%). No clinically significant bleeding events or diarrhoea were reported in the study cohort. Dose reduction due to treatment-related toxicities was required in 11 patients (11.8%), while temporary treatment interruption occurred in eight patients (8.6%). Permanent treatment discontinuation secondary to adverse events was observed in six patients (6.5%) (Table 2).

**Table 2:** Adverse events associated with BTK inhibitors in patients with CLL (N = 93). CLL: chronic lymphocytic leukaemia; BTK: Bruton's tyrosine kinase

Adverse Event	n (%)
Anaemia	6 (6.5)
Neutropenia	6 (6.5)
Thrombocytopenia	5 (5.4)
Fatigue	4 (4.3)
Arthralgia	6 (6.5)
Hypertension	4 (4.3)
Atrial fibrillation/flutter	2 (2.2)
Infections	4 (4.3)
Bleeding events	0
Diarrhea	0
Treatment Modifications	
Dose reduction	11 (11.8)
Treatment interruption	8 (8.6)
Permanent discontinuation	6 (6.5)

## Discussion

**Figure 10:**
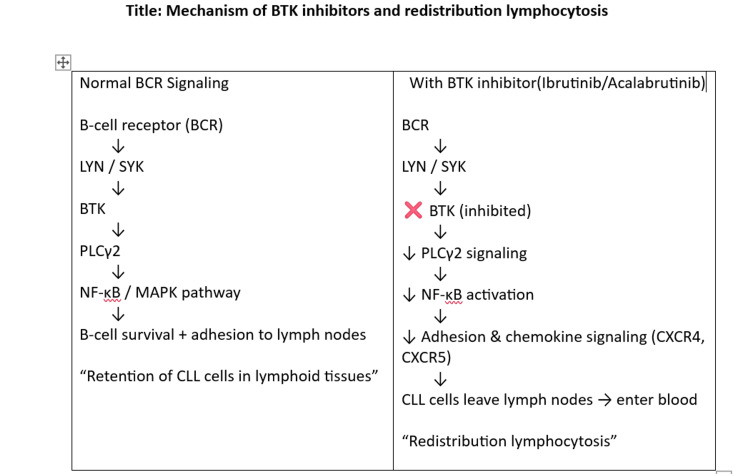
Mechanism of action of Bruton's tyrosine kinase (BTK) inhibitors and redistribution lymphocytosis. In normal B-cell receptor (BCR) signalling, activation of BTK leads to downstream signalling through PLCγ2 and NF-κB pathways, promoting B-cell survival and retention within lymphoid tissues. BTK inhibitors such as ibrutinib and acalabrutinib block this pathway, resulting in impaired adhesion and chemokine signalling. Consequently, malignant B cells redistribute from lymph nodes into the peripheral blood, leading to transient lymphocytosis. PLCγ2: phospholipase C gamma 2; NF-κB: nuclear factor kappa B; LYN: LYN proto-oncogene; SYK: spleen tyrosine kinase; MAPK: mitogen-activated protein kinase; CXCR4: C-X-C chemokine receptor type 4; CXCR5: C-X-C chemokine receptor type 5; CLL: chronic lymphocytic leukemia Image Credit: The schematic was created by the authors using Microsoft Word (Microsoft Corp., Redmond, WA, USA).

The findings of the present study are consistent with previously reported data demonstrating the efficacy of BTK inhibitors in both treatment-naïve and relapsed/refractory CLL. However, adverse events such as atrial fibrillation, bleeding, and hypertension remain clinically relevant, particularly with first-generation agents. The improved selectivity of second-generation BTK inhibitors may contribute to a more favourable safety profile, which is reflected in our observations. These findings highlight the importance of individualised treatment selection, especially in patients with comorbidities. This study reinforces the transformative role of BTK inhibitors in CLL management. Hematologic recovery rates and time to response were comparable to major Indian and international trials. Acalabrutinib demonstrated a consistently better safety profile, aligning with findings from ELEVATE-TN and ELEVATE-RR trials. The mechanism of BTK inhibition and redistribution lymphocytosis in CLL is illustrated in Figure 10.

In our study, the median age of our patient population was 57 years. The findings closely correlate with other published Asian and Indian data on CLL. In our study, most patients were male and younger than typical Western CLL cohorts. Hematologic recovery rates were high, with normalisation of haemoglobin, platelet count, and leukocyte count seen in the majority within four to seven months of therapy. The overall median PFS was 14 months.

Acalabrutinib is a next-generation, irreversible BTK inhibitor approved for the treatment of CLL and small lymphocytic lymphoma (SLL). Compared with ibrutinib, it demonstrates greater selectivity for BTK and a shorter plasma half-life, which may contribute to fewer off-target adverse effects.

Comparison between ibrutinib and acalabrutinib

Safety remains a key differentiator when selecting among BTK inhibitors. ELEVATE-RR demonstrated non-inferior efficacy with lower rates of atrial fibrillation/flutter and hypertension for acalabrutinib compared with ibrutinib. This resulted in fewer discontinuations due to adverse events.

Kaplan-Meier analysis demonstrated largely overlapping PFS curves between patients receiving ibrutinib and acalabrutinib, suggesting comparable short-term survival outcomes between the two BTK inhibitors. Although acalabrutinib demonstrated a numerically lower frequency of treatment interruptions and cardiovascular adverse events, no statistically significant difference in PFS was observed between the groups during the available follow-up period [8].

Emerging comparative effectiveness studies and updated National Comprehensive Cancer Network (NCCN) guidelines increasingly support second-generation BTK inhibitors, including acalabrutinib and zanubrutinib, as preferred treatment options based on both efficacy and safety considerations [9].

Ibrutinib remained widely used but demonstrated higher frequencies of adverse events such as anaemia, fatigue, arthralgia, and diarrhoea. Acalabrutinib appeared to be associated with improved tolerability and treatment continuation; however, these findings should be interpreted cautiously, given the observational and non-randomised study design [10].

Notably, our findings mirror global observations that covalent BTK inhibitors mitigate the adverse prognostic impact of TP53 aberrations and unmutated immunoglobulin heavy chain variable region (IGHV), although India‑specific genetic datasets remain relatively small and should be expanded to confirm effect sizes locally. ELEVATE‑TN’s long‑term analysis demonstrated maintained PFS benefit with acalabrutinib‑based therapy irrespective of unmutated IGHV or TP53 disruption; likewise, the ALPINE program reported persistent PFS superiority with zanubrutinib in relapsed/refractory chronic lymphocytic leukaemia (R/R CLL), including del(17p)/TP53‑mutated subsets [11]. Limited access to molecular profiling (TP53/IGHV) remains a major challenge in the Indian setting, impacting treatment stratification. The real-world nature of this study highlights practical constraints such as inconsistent drug availability under public insurance schemes, affecting continuity of care.

This study reinforces the transformative role of BTK inhibitors in CLL management. Hematologic recovery rates and time to response were comparable to major Indian and international trials. Acalabrutinib demonstrated a consistently better safety profile, aligning with findings from ELEVATE-TN and ELEVATE-RR trials.

Limitations

The study had certain limitations, such as unequal distribution between the ibrutinib and acalabrutinib arms due to drug availability and limited access to TP53/IGHV testing due to cost-effectiveness. The follow-up period was short, limiting the assessment of OS, and government insurance restrictions caused treatment interruptions for ibrutinib.

## Conclusions

Both ibrutinib and acalabrutinib demonstrated clinically meaningful responses in patients with CLL in this real-world cohort. Acalabrutinib appeared to be associated with a favourable tolerability profile and fewer treatment interruptions; however, given the retrospective observational design and limited sample size, definitive comparative conclusions between the two BTK inhibitors cannot be established. Larger prospective studies with longer follow-up are warranted to further evaluate long-term efficacy and safety outcomes.

## References

[1] Hallek M (2025). Chronic lymphocytic leukemia: 2025 update on the epidemiology, pathogenesis, diagnosis, and therapy. Am J Hematol.

[2] Campo E, Cymbalista F, Ghia P (2018). TP53 aberrations in chronic lymphocytic leukemia: an overview of the clinical implications of improved diagnostics. Haematologica.

[3] Nayyar M, Menezes RC, Ailawadhi S, Parrondo RD (2025). Chronic lymphocytic leukemia: novel therapeutic targets under investigation. Cancers (Basel).

[4] Frustaci AM, Deodato M, Zamprogna G, Cairoli R, Montillo M, Tedeschi A (2023). Next-generation BTK inhibitors in CLL: evolving challenges and new opportunities. Cancers (Basel).

[5] Ran F, Liu Y, Wang C (2022). Review of the development of BTK inhibitors in overcoming the clinical limitations of ibrutinib. Eur J Med Chem.

[6] Brown JR, Byrd JC, Ghia P (2022). Cardiovascular adverse events in patients with chronic lymphocytic leukemia receiving acalabrutinib monotherapy: pooled analysis of 762 patients. Haematologica.

[7] Tejaswi V, Lad DP, Jindal N (2020). Chronic lymphocytic leukemia: real-world data from India. JCO Glob Oncol.

[8] Byrd JC, Hillmen P, Ghia P (2021). Acalabrutinib versus ibrutinib in previously treated chronic lymphocytic leukemia: results of the first randomized phase III trial. J Clin Oncol.

[9] Burger JA, Tedeschi A, Barr PM (2015). Ibrutinib as initial therapy for patients with chronic lymphocytic leukemia. N Engl J Med.

[10] Sharman JP, Egyed M, Jurczak W (2025). Acalabrutinib-obinutuzumab improves survival vs chemoimmunotherapy in treatment-naive CLL in the 6-year follow-up of ELEVATE-TN. Blood.

[11] (2026). NCCN Clinical Practice Guidelines in Oncology: chronic lymphocytic leukemia/small lymphocytic lymphoma. Version 2.2023. https://www.nccn.org/guidelines/guidelines-detail?category=1&id=1478.

